# Does altercentric interference rely on mentalizing?: Results from two level-1 perspective-taking tasks

**DOI:** 10.1371/journal.pone.0194101

**Published:** 2018-03-22

**Authors:** Julia Marshall, Anton Gollwitzer, Laurie R. Santos

**Affiliations:** Psychology, Yale University, New Haven, Connecticut, United States of America; University College London, UNITED KINGDOM

## Abstract

Considerable debate has focused on whether adults possess an implicit system for representing others’ mental states. Some argue that people automatically represent the perspective of others using evidence from altercentric interference—cases in which another agent’s perspective affects the speed with which one can report one’s own perspective. Others have argued that altercentric interference is not always specific to social stimuli and thus may represent a simpler process such as submentalizing. To distinguish between these positions, Study 1 developed a novel measure of altercentric interference—a “sandbox” measure—that allowed us to more sensitively assess altercentric interference across social and non-social conditions. We replicated previous findings showing that participants experience both egocentric and altercentric interference, but we found that these effects emerge equally in social and non-social conditions. To further test whether altercentric interference emerges in social perspective-taking situations, Study 2 conducted a conceptual replication of a study which used a novel “goggle” paradigm to assess whether individuals implicitly represent others’ perspectives. Although we failed to find evidence of altercentric interference in response times, participants’ accuracy reflected the possibility of interference from others’ perspectives. We argue that these findings provide support for the idea that altercentric interference in response to social stimuli (an avatar) is driven by perspective-taking mechanisms, while such interference in response to non-social stimuli (an arrow) is driven by attention-cuing mechanisms.

## Introduction

Navigating the social world requires complex computations about the often variable contents of other people’s minds. Indeed, other people’s minds can include anything from simple perspectives, such as what one sees, to complex belief states, such as thinking something is false. Despite widespread agreement that adults—and even infants as young as 15 months of age [1–2]—appear to possess capacities for understanding other people’s perspectives and mental states, much debate exists around the nature of these capabilities. Do humans rapidly and efficiently compute other people’s perspectives, goals, and belief states? Or does such a capacity emerge from more effortful, deliberative thinking?

One prominent answer to this question is that humans have two different “theory of mind” (ToM) systems: an implicit and early emerging system and a second more effortful system, that develops later in life [3–4]. In support of this idea, researchers have found that infants in the second year of life appear to represent others’ false beliefs when tested using implicit looking time measures [1, 5], even though they perform poorly on false belief tests that require explicit, verbal responses until around four years of age [6]. In addition, researchers have observed that non-neurotypical populations like those with autism spectrum disorder (ASD) show different performance when tested on implicit and explicit ToM tasks. ASD individuals show less evidence of implicit mentalizing than neurotypical individuals [7–8], although both ASD and non-ASD individuals perform similarly on explicit verbal ToM tasks.

Taken together, this work has led many researchers to argue that humans possess two systems for representing others’ mental states, one of which computes others’ mental states implicitly, rapidly, and efficiently [9] and the other of which computes others’ mental states in a deliberative manner. Hereafter, we will refer to this view as the *implicit mentalizing* view: the claim that at least some of our mentalizing occurs rapidly and without cognitive effort [3–4].

Several researchers have argued against this implicit mentalizing position with an alternative hypothesis: there is in fact no implicit, early emerging system for representing others’ beliefs. Under this so-called *submentalizing account*, effects assumed to result from implicit mentalizing instead result from experimental artifacts [10–11], such as simple attentional cueing [12]. Proponents of this submentalizing position argue that perspective taking and belief representation result *only* from a cognitively-effortful and later emerging system [13].

The debate around whether the implicit mentalizing or submentalizing hypotheses better captures humans’ ToM capacities has received significant attention in recent years [9]. Much of this debate has centered around contested findings on a dot perspective-taking task, originally developed by Apperly and colleagues [14–15]. In this task, participants are asked to count the number of dots on a screen. Importantly, an avatar is also present on the screen when the dots are revealed. Critically, the avatar sees a number of dots that is either the same as or less than the number of dots that the participant sees. Participants are then asked to verify either their own perspective or the avatar’s perspective. In line with previous work on the ‘curse of knowledge’ [16], participants take longer to report the number of dots the avatar sees when the number of dots the participant sees is different. That is, participants experience *egocentric interference* on this task: participants’ ability to verify what the avatar sees is affected by participants’ own perspective. More importantly for the debate surrounding implicit mentalizing, however, participants also take longer to report how many dots they themselves see when the avatar sees a different number of dots. This second form of error, which has been central to mounting a case for the implicit mentalizing hypothesis, is characterized as *altercentric interference*: participants’ ability to report their own perspective is affected by the perspective of the avatar.

A number of critics have challenged the claim that altercentric interference on the dot perspective-taking task provides evidence for an implicit mentalizing system. Indeed, several scholars have argued that similarly strong altercentric interference effects emerge in an analogous task that involves no agent (and thus cannot result from mentalizing or perspective taking). For example, Heyes [12] has demonstrated that altercentric interference effects emerge in the dot perspective-taking task when the avatar is replaced with an arrow [11]. This finding has been leveraged to argue that humans do not effortlessly compute others’ perspectives; rather, altercentric interference effects arise because of attentional cueing and are not specifically tuned to social scenarios as the implicit mentalizing hypothesis would predict.

Which of these two interpretations is correct, however, has elicited a considerable amount of debate in recent years. Much of this work has focused on assessing the domain-specificity of the altercentric interference effect: does altercentric interference indeed emerge in situations that are *specifically social*, as the implicit mentalizing account would predict? In one study, Nielsen, Slade, Levy, and Holmes [17] found that altercentric errors did emerge in a perspective-taking task in social (an agent), semi-social (an arrow), and non-social (a dual-colored block) conditions, but the magnitude of these altercentric interference effects were most pronounced in the social condition. They also found that altercentric interference effects correlate with self-reported perspective taking and empathy for the social condition only. In a second line of work, Drayton, Boothby, Santos, and Dunham [18] found that altercentric interference effects are specifically susceptible to social biases; Drayton and colleagues found that White participants spontaneously took the perspective of a White avatar more frequently than that of a Black avatar. Taken together, these findings suggest that the magnitude of altercentric interference observed on a given task is affected by socially relevant variables, such as individual differences in people’s social biases and the social category of an avatar.

In contrast to these findings, a sizable amount of counter-evidence demonstrates that altercentric interference can occur in non-social situations and with populations who lack social aptitude. For instance, Cole, Atkinson, Le, and Smith [19] found that participants still demonstrate altercentric inference even when the avatar cannot see the dots because of an intervening wall (although see [20]). Additionally, using a slightly different task to assess perspective taking, Santiesteban, Shah, White, Bird, and Heyes [21] found that performance was unaffected by whether a human or camera served as an avatar. They also found that populations with known deficits in mentalizing, namely ASD participants, performed similarly to neurotypical participants on an implicit perspective-taking task. Finally, Wilson, Soranzo, and Bertamini [22] elicited consistent rapid perspective-taking effects while systematically manipulating the social relevance of the scene, suggesting that perspective taking is not exclusively tuned to social stimuli. These results hint that participants may not be computing the avatar’s perspective during perspective taking tasks, but rather are exhibiting an interference effect because of more domain general attentional cues.

To specifically test this deflationary attentional account, Furlanetto, Becchio, Samson, and Apperly [23] adapted the original dot perspective taking task so that a human agent’s attentional cues were always present, but manipulated whether the human could see. To do so, they presented participants with an avatar that sometimes wore opaque or translucent goggles. Participants showed altercentric interference when the avatar was wearing translucent goggles or no goggles but not when the avatar wore opaque goggles (i.e., when the avatar could not see). This finding provides strong evidence that altercentric interference is affected by cues related to perspective taking, thereby providing more support for the implicit mentalizing hypothesis. A recent paper, however, failed to find similar effects in a replication attempt [24], making it difficult to determine the robustness of Furlanetto and colleagues’ [23] effects.

To date, researchers have used numerous methodological adaptations to Samson et al., [14]’s original dot perspective-taking task to test whether altercentric interference results from implicit mentalizing per se or from more domain-general submentalizing processes. These methodological changes have included varying the social nature of the stimuli, examining how individual differences on other social measures affect altercentric interference, and altering elements of the experimental scene to reduce confounds across consistent and inconsistent trials.

In the present paper, we seek to contribute to this burgeoning body of research by conducting two studies aimed at varying other important features of the dot perspective-taking task. In Study 1, we developed a *novel dependent measure* of altercentric interference, one that can more sensitively detect possible differences in altercentric interference for social and non-social stimuli. By adopting a potentially more sensitive measure of accuracy, we hope to better capture whether participants’ performance differs for social versus non-social stimuli. In Study 2, we conducted a *conceptual replication* of what we thought was the most convincing finding in support of the implicit mentalizing position to date—Furlanetto et al.’s [23] finding that people exhibit altercentric interference when the avatar in the dot-perspective taking task is wearing translucent goggles but not when the avatar is wearing opaque goggles (i.e., when the avatar cannot see). Since one recent paper has reported failing to replicate this effect [24], we felt an additional attempt to find an effect similar to that of Furlanetto and colleagues’ [23] would provide important insight into the robustness of this finding.

## Study 1

In our first study, we developed a continuous dependent measure to more sensitively test levels of altercentric interference across social and non-social stimuli. Altercentric interference is thought to occur when another agent’s perspective interferes with the representation of one’s own perspective. In this way, true altercentric interference should affect the *accuracy* of one’s perspective judgments. Unfortunately, researchers have tended not to use accuracy as a measure of altercentric interference since this measure is often binary (e.g., correct vs. incorrect).

To use a continuous measure, researchers have historically measured altercentric interference by using differences in *response time*, specifically whether people take longer to report their own perspective when an avatar has a different versus similar perspective. Researchers then compare these continuous response time differences across different conditions (e.g., social vs. non-social) to see how different social variables affect levels of altercentric interference. Although response time measures helpfully provide a continuous measure of altercentric interference, such measures are potentially problematic in that different kinds of factors can lead to increased response time on perspective-taking tasks.

With this in mind, we developed a novel continuous measure of the accuracy of participants’ egocentric perspective reports. To do so, we borrowed a continuous measure originally developed to test fine-grained differences in the accuracy of children’s mental state representations—a “sandbox” measure [25–26]. Sommerville and colleagues [26] developed this sandbox measure as a more continuous measure of children’s accuracy in a false belief task [27]. In a typical false belief task, child participants see one puppet, Sally, place a toy in a certain location and leave the room. A second puppet, Anne, then comes into the room and changes the location of the toy. Upon Sally’s return, children are typically asked in which of the two locations Sally will look for her toy. Historically, children’s accuracy on this task was measured in a binary way (e.g., correct or incorrect).

Sommerville et al. [26] provided a major innovation on this task by changing this binary measure of accuracy into a more continuous one. To do so, they modified the standard task so that that Sally hid her toy in one side of a long sandbox rather than one of two locations. Anne then moved the toy and hid it inside the sand on the other side of the sandbox. Children were then asked where in the sandbox Sally will look for her toy. Using this more continuous sandbox measure, Sommerville and colleagues [26] found that the extent of children’s errors in search location decreases as children get older.

In Study 1, we apply the logic of this continuous sandbox measure to the dot perspective-taking task. Specifically, we allowed participants to report how many dots they saw from their own and an avatar’s perspective on a number line. We could then test whether the location participants indicate on the number line differs depending on whether an avatar has a different versus similar perspective. Further, we could compare these continuous number line differences across different conditions (e.g., social vs. non-social) to see how different conditions affect levels of altercentric interference. Our novel continuous accuracy measure also allowed us to examine whether altercentric interference emerges for dependent measures outside the typical ones (i.e., response times), which also aids in understanding the generalizability of altercentric interference effects.

In adopting this measure, two possibilities emerge: one possible hypothesis is that the magnitude of participants’ errors may decrease as a function of the sociality of the stimulus. That is, participants may make greater errors for human avatars because participants are indeed representing their contents of another agent’s mind in a more differentiated way than for arrows. Another possibility is that the magnitude of participants’ errors will not differ as a function of the sociality of the stimulus.

To best maximize our use of this new number line measure, we increased the *total* maximum number of dots seen by the participant in our task. In a typical dot perspective-taking study [14–15], participants see between 0 and 3 dots. We worried that this small range of numbers would not adequately capture variance in participants’ number line choices. Rather than arbitrarily varying the numbers seen by the participant and avatar, we drew on research from the number cognition literature that adults are better able to discern the difference between two numbers when the ratio between the two numbers is small [28]. Specifically, individuals are better able to detect differences between numbers as the ratio between the numbers decreases. With this in mind, we expanded the numbers of dots participants saw in our task (maximum of 12 total dots) and varied the ratio between the participant’s and avatar’s perspective.

### Method

The research presented in the current manuscript was conducted under the approved protocol: 1503015578: Theory of Mind Applied to Different Social Categories (the author’s institution).

#### Participants

We tested adults (*N* = 49) via Amazon Mechanical Turk (MTurk). Three participants failed to correctly input an ID number when directed to an external link to complete the task and thus we were unable to collect their demographic information. A power analysis indicated that with this sample size, we would have had 92.91% power to detect an effect of moderate size (Cohen’s *d* = .50). Among the remaining 46 participants, 27 were male and 19 were female, ranging in age from 20 to 61 years of age (*M* = 30.65, *SD* = 8.59).

#### Procedure and materials

The procedure consisted of three elements: (1) Instructions, (2) Practice Trials, and (3) the Dot Perspective-Taking Task.

(1) Instructions: Participants first entered the task via M-Turk and viewed instructions on a Qualtrics page. Specifically, we told participants:

In this experiment, you will see several images that depict either a man looking at squares or an arrow oriented toward squares. Sometimes the man or the arrow will be looking/pointing to the left and other times the man or arrow will be looking/pointing to the right. Before seeing an image, you will be prompted to attend to a) how many squares you see indicated by the prompt YOU; b) how many squares the man sees indicated by the prompt HIM; or c) how many squares the arrow is oriented towards indicated by the prompt ARROW. You will then see a rating scale. You should click on the number line where you believe the number is located that corresponds with the perspective you were asked to attend to.

We then showed participants practice images with correct answers to ensure that participants understood the directions. Specifically, participants saw an image where the participant and the avatar both saw 2 squares. Underneath the image read: “For instance, if you were asked to attend to YOU, you would indicate 2 on the rating scale. If you were asked to attend to HIM, you would also indicate 2.” They also saw two other practice images: one where an avatar was oriented toward a different number of squares than depicted on the whole screen and one where the arrow was oriented toward a different number of squares than depicted on the whole screen. Underneath these images read: “In other circumstances, the task may be more challenging. Here, if you were asked to attend to YOU, the correct response would be 2. If you were asked to attend to [HIM/ARROW], the correct response would be 1.” Participants then acknowledged they understood the task and clicked a link to enter the experiment.

(2) Practice trials: The participant then entered their ID number into an external task and participated in ten practice trials to adjust to the nature of the experiment. These trials comprised ten trials that included a random sampling of experimental trials from the experimental dot perspective-taking task. Upon completing those trials, the participant was told that the practice trials were complete and that they were to begin the test session. They were told to complete the entire task in one sitting in a quiet setting.

(3) Dot perspective-taking task: The dot perspective-taking task was based off of theSamson et al. [14] study. Each trial consisted of a person standing in the middle of a room oriented either to the left or the right. All the trials systematically differed from one another. In configuring the experiment, we manipulated perspective (self, other), consistency (consistent, inconsistent), scene sociality (human, arrow), and ratio (small, large).

As in previous dot perspective-taking task studies [14–15], we varied which *perspective* participants were prompted to attend to across the trials. On half the trials, participants were instructed to pay attention to their own perspective (“self” trials) by the prompt “YOU”. On the other half, participants were instructed to attend to the arrow or avatar (“other” trials) by the prompt “HIM” or “ARROW”. As noted previously, we also manipulated *consistency*. Participants saw the same number of squares as avatar/arrow for half of trials (consistent trials), whereas participants saw a different number of squares than the avatar/arrow for the other half of trials (inconsistent trials).

We made two modifications to the original task: a *scene sociality manipulation* and a *ratio manipulation*. For the *scene sociality* manipulation, we varied the sociality of the avatar. Specifically, we used either a social stimulus (a human avatar) or a non–social stimulus (an arrow). For the *ratio manipulation*, we varied the difference in the ratio of squares seen from the perspective of the avatar and the participant; we used both a small ratio difference (.25) and a large ratio difference (.50). Because we did not want participants to always see the same ratio condition with the same set of squares (e.g., the other’s perspective was always 1 and the participant’s perspective was always 4 for the small ratio condition), we also varied *the quantity of squares* on the screen to allow for two quantities for each ratio. In doing so, the total amount of squares on the screen varied from 2, 4, 6, or 12. The small ratio was always depicted in the quantity 4 (1 as the other’s perspective and 4 as the participant’s perspective) or 12 (3 as the other’s perspective and 12 as the participant’s perspective). The large ratio (.50) was always depicted with the quantities 2 (1 as the other’s perspective and 2 as the participant’s perspective) or 6 (3 as the other’s perspective and 6 as the participant’s perspective). We controlled the amount of pixels the black square occupied across all ratio trials.

**Fig 1:**
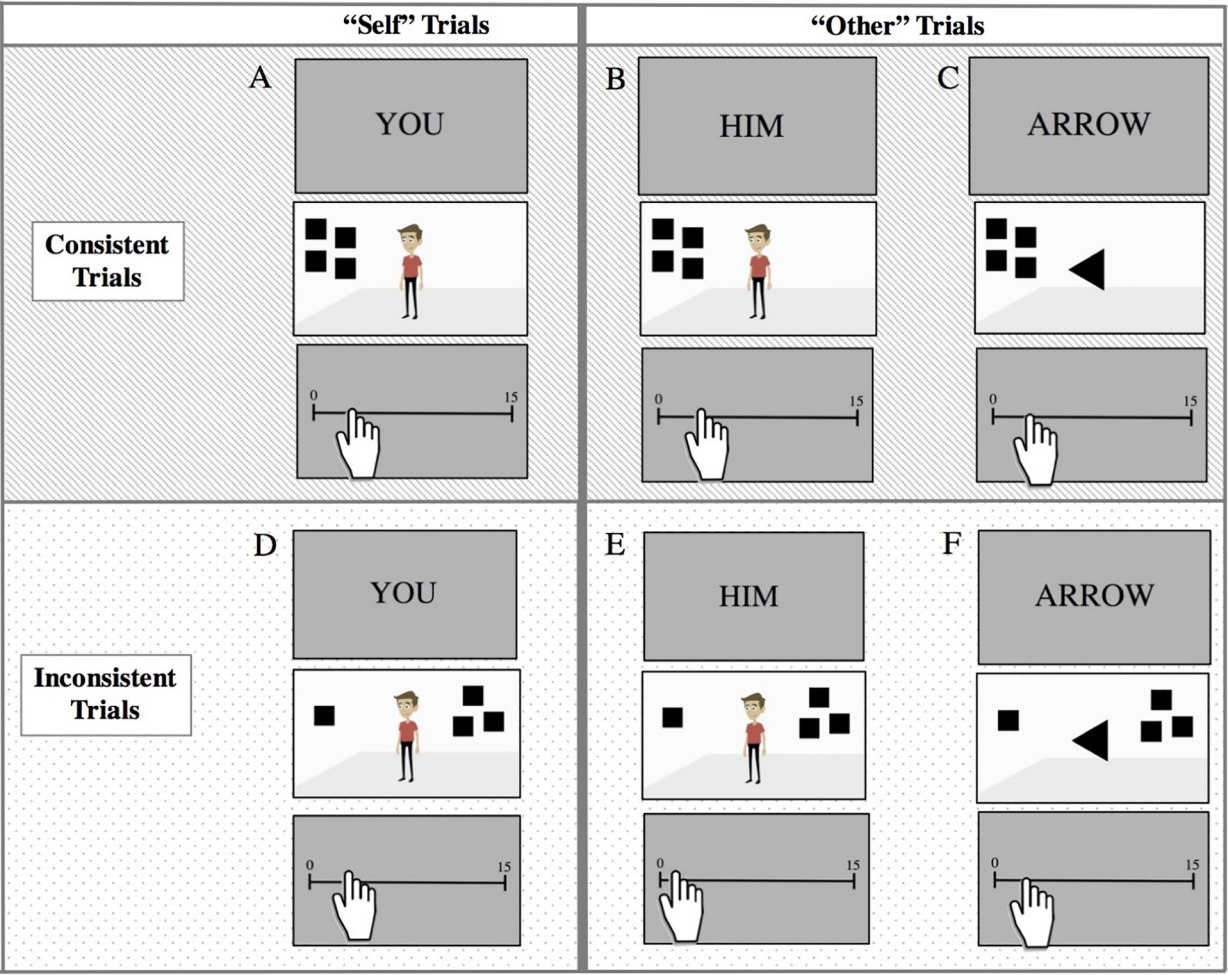
Examples of trials from Study 1. Depicted here are examples of consistent and inconsistent trials across “Self”/“Other” and social/non-social trials: (A) Self/Consistent/Social trial—Correct Answer: 4; (B) Other/Consistent/Social trial—Correct Answer: 4; (C) Other/Consistent/Non-social trial—Correct Answer: 4; (D) Self/Inconsistent/Social trial—Correct Answer: 4; (E) Other/Inconsistent/Social trial—Correct Answer: 1; (F) Other/Inconsistent/Non-social trial—Correct Answer: 1.

Each trial began with a fixation cross (presented for 750 milliseconds) preceded by a perspective prompt (i.e., “self” or “other” trials; presented for 750 milliseconds; see Fig 1). After the perspective prompt, the participant saw a stimulus image for 1500 milliseconds. The participant then saw a sliding scale anchored on the right with a 15 and on the left with a 0 for 1500 milliseconds. Note that no intermediate numbers were displayed as to allow for participants to quickly respond to the task without having to read more than two number labels. The participant was then asked to perform a mouse click on the continuous scale to indicate their answers of how many squares. The order of the stimuli was fixed for all participants a priori via a random number table. We adhered to the random ordering except when the ordering indicated consistent or inconsistent trials occurring more than 3 consecutive times.

### Results

#### Data preparation

Prior to conducting any analyses, error values were calculated by subtracting the participant’s actual number line response from the correct response on every trial. Negative values indicated that the participant clicked lower than the true value on the number line (negative errors), whereas positive values indicated that the participant clicked higher than the true value on the number line (positive errors). In the case of consistent trials, positive and negative errors represent mistaking the true value seen. In inconsistent “self” trials, negative errors represent altercentric errors because the avatar/arrow always saw less than the overall number of squares depicted on the screen. Positive errors represent overestimation of the total amount of dots seen by the participant. In inconsistent “other” trials, negative errors represent underestimating the true value from the perspective of the avatar/arrow. Positive errors in the inconsistent “other” trials represent egocentric errors. This is because the avatar always saw less, therefore any positive correction represents a pull in the direction of participant’s perspective. Finally, because each participant also responded to each condition twice, responses to both conditions were averaged to render a mean error for each condition for every participant. We also collected and analyzed response time data [see S1 File for full information].

#### Errors

**Fig 2:**
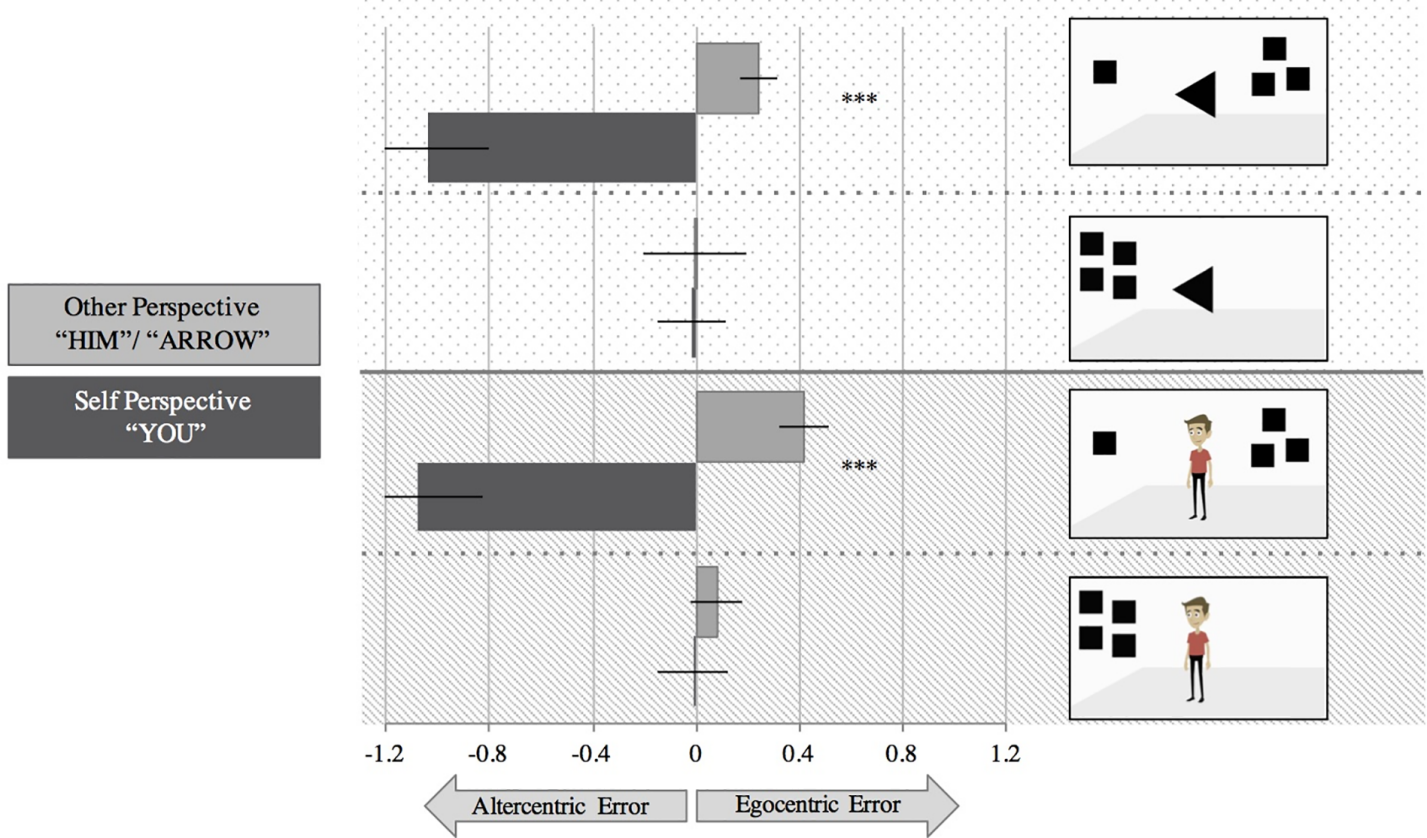
Depiction of the Perspective x Consistency x Sociality interaction. Error bars represent ±1 SEM; *** *p* < .001.

We ran a 2 (Perspective: You, Him/Arrow) x 2 (Consistency: Consistent, Inconsistent) x 2 (Sociality: Human, Arrow) x 2 (Ratio: Small, Large) repeated measures analysis of variance (ANOVA). Here, the Perspective x Consistency x Sociality x Ratio interaction was not significant, *F*(1, 48) = 2.82, *p* = .100, η_p_^2^ = .056; individuals’ errors did not differ as a function of small or large ratios. Because this four-way interaction was not significant, we collapsed across ratio. We then ran a 2 (Perspective: You, Him/Arrow) x 2 (Consistency: Consistent, Inconsistent) x 2 (Sociality: Human, Arrow) repeated measures ANOVA. Again, we did not find a significant Perspective x Consistency x Sociality interaction, *F*(1, 48) = .38, *p* = .542, η_p_^2^ = .008. As Fig 2 illustrates, there were no consistent differences in errors between the arrow and human condition within the perspective and consistent trials.

We therefore collapsed across sociality to see whether there was a Perspective x Consistency interaction. There was a significant main effect of Consistency, *F*(1, 48) = 13.01, *p* = .001, η_p_^2^ = .213. Participants made greater errors in the inconsistent trials, *M* = -.36, *SD* = 1.02, compared to the consistent trials, *M* = .01, *SD* = .90. We also found a significant main effect of Perspective, *F*(1, 48) = 23.84, *p* < .001, η_p_^2^ = .332; specifically, participants made more altercentric errors on “self” trials, *M* = -.54, *SD* = 1.22, compared to in “other” trials, *M* = .18, *SD* = .69, in which they made egocentric ones. Importantly, there was a significant Perspective x Consistency interaction, *F*(1, 48) = 29.76, *p* < .001, η_p_^2^ = .383. When participants were asked about their own perspective, they made greater errors in inconsistent trials, *M* = -1.06, *SD* = 1.54, compared to consistent trials, *M* = -.02, *SD* = .91; *t*(48) = 5.62, *p* < .001. Similarly, when participants were asked about the avatar’s or arrow’s perspective, they made greater errors in the inconsistent trials, *M* = .33, *SD* = .50, compared to the consistent ones, *M* = .04, *SD* = .90; *t*(48) = 2.23, *p* = .03.

Because small samples tend to lead to increased Type II error, we also tested for null effects using Bayesian statistics using JASP (https://jasp-stats.org/). These analyses compare models according to a null hypothesis and an alternative hypothesis for best fit [29]. Using this technique, we compared the strength of the consistency effect in the human condition compared to the arrow condition. We tested for a null effect because past research has consistently found altercentric effects both for avatars as well as arrows [11, 12, 19, 21]. The Cauchy prior–a hypothesis effect size (Cohen’s *d*) distribution–was the default in the JASP software (.707). The estimated Bayes factor suggested that the data were 6.34: 1 in favor of the null hypothesis—this was categorized as a strong effect. That is, it is 6.34 times more likely that a null effect exists such that the observed altercentric effects are no stronger in the human condition than the arrow one. Overall, these findings indicate that individuals make altercentric and egocentric errors (in terms of accuracy) in the presence of both social and non-social stimuli.

It is worth nothing that we also examined whether there was an effect of participant gender. In Samson et al. [14]’s study, they matched the gender of the avatar to that of the participants’ most likely to account for mentalizing differences in cases where the gender of the participant differs from the avatar. Here, the gender of the avatar was always male. Importantly though, we did not find an interaction effect of gender, *p* = .552.

### Discussion

Study 1’s primary aim was to assess whether changing the dependent measure of the typical dot perspective-taking task from response times to a more sensitive, continuous measure of accuracy would reveal stronger altercentric interference effects for social stimuli compared to non-social stimuli. In other words, would individuals pull their perspective indication on a number line toward that of the avatar’s more so than in cases when the arrow was present? We did not find evidence of this possibility. Although we replicated the effect documented in Samson et al. [14] that individuals make both egocentric and altercentric errors in the dot perspective-taking task, we also found that participants made these sorts of errors for *both* non-social stimuli (i.e., arrows) and social stimuli (i.e., avatars).

In this way, our results align with previous work showing that altercentric interference is not specifically social in nature [11–12]. Importantly, Study 1 builds on these previous findings in that we observed no difference between social and non-social conditions even when our more sensitive sandbox measure is used. Previous evidence that altercentric interference is not domain-specific has used response time measures, which may be problematic because they are particularly susceptible to a myriad of factors. Our results using a more sensitive sandbox task observe a similar pattern as previous response time studies: participants had comparable levels of errors in the arrow condition and the avatar condition. This finding therefore fits with other research suggesting that altercentric interference effects may result from submentalizing processes. Our findings also broadly align with literature indicating that individuals do not rapidly represent the minds of others [11, 12, 19, 21, 22].

## Study 2

At first glance, the results of Study 1 did not indicate support for the implicit mentalizing position–participants were equally inaccurate when the interfering stimulus was social versus non-social. However, the findings are consistent with two possibilities. For one, it is plausible that altercentric errors emerge for both the arrow and the avatar because of strictly attentional cues. Second, it is also possible that errors that appear like altercentric errors emerge in the arrow case because of attentional cueing, whereas the altercentric errors emerge in the avatar case because of rapid perspective-taking mechanisms. Study 1 cannot discern between these two possibilities.

In light of this, we opted to conduct a conceptual replication of a previous study to shed light on which account is correct. Specifically, we attempted to replicate Furlanetto et al. [23]’s study which provides some of the strongest evidence to date that the adults implicitly represent the perspective of others. As reviewed above, Furlanetto et al. [23] varied whether an avatar could see the dots in the dot perspective-taking task by making the avatar “blind” or not. Specifically, they manipulated whether an avatar could see the dots by having the avatar wear opaque goggles or not. Note that this design, by making the avatar “blind” or not, varies perspective-taking cues independently of attentional cuing. These researchers found that participants made altercentric errors only in the cases when the avatar wore translucent or no glasses (i.e., was not blind), but not when the avatar wore opaque ones (i.e., was blind). These findings provide evidence that altercentric interference effects arise because of perspective-taking mechanisms and not attentional-cuing mechanisms.

It is also worth mentioning that we did not utilize the sandbox measure in this study because the primary aim was to replicate Furlanetto et al. [23]’s findings and not to further examine whether adopting a continuous accuracy measure may play a role in interpreting altercentric interference effects.

### Method

#### Participants

We tested 45 individuals on Amazon Mechanical Turk. Ten participants were excluded for failing comprehension checks and one participant was excluded for not responding to all trials. The final sample thus included 34 participants ranging in age from 22 to 59 years (*M* = 36.34, *SD* = 9.25). Using the effects of Furlanetto et al. [23] as a benchmark (the smallest effect of importance was the inconsistency effect in the eyes condition, *t*(16) = 2.32, Cohen’s *d* = 0.56), a power analysis revealed that we would have 97% power to detect an effect of this size in our sample.

#### Procedure and materials

The procedure was similar to Furlanetto et al. [23] with several important differences. The procedure consisted of four elements: (1) Instructions, (2) Seeing Induction, (3) Practice Trials, (4) Dot Perspective-Taking Task.

(1) Instructions. Participants first entered the task via M-Turk and viewed instructions on Qualtrics. Specifically, we told participants:

In this experiment, you will see several images that depict a man looking at circles. Sometimes the man will be looking to the left and other times the man will be looking to the right. Before seeing an image, you will be prompted to attend to a) how many circles you see indicated by the prompt YOU, or b) how many circles the man sees indicated by the prompt HIM. You will then see a number, ranging from 0 to 3, and then an image. When you see an image, a) click A if the number matches the number of circles the person you were prompted to attend to saw, or b) click L if the number does not match the number of circles the person you were prompted to attend to saw.

Participants then saw several example trials to ensure they understood the nature of the task, as was done in Study 1. For instance, participants saw a consistent trial in which both the participant and the avatar saw three dots. Participants were told: “If you were prompted with YOU and then 3, you would indicate yes (A). If you were prompted with YOU and then 2, you would indicate no (L). If you were prompted with HIM and then 3, you would indicate yes (A). If you were prompted with HIM and then 2, you would indicate no (L).” They also saw an example of an inconsistent trial with similar guidance. Participants were then asked two comprehension questions in which they saw a consistent and inconsistent trial. Specifically, they were shown an inconsistent trial when the participant’s perspective was 2 and the avatar’s was 1. Participants were asked, “If you were prompted with YOU and 2, what would you click: A or L? (Answer: A)” They were also shown a sample trial in which an avatar did not see any dots and the participant saw 2. They were prompted, “If you were prompted with HIM and 2, what would you click: A or L? (Answer: L)” Participants had to respond correctly to these two questions to be included in final analyses.

**Fig 3:**
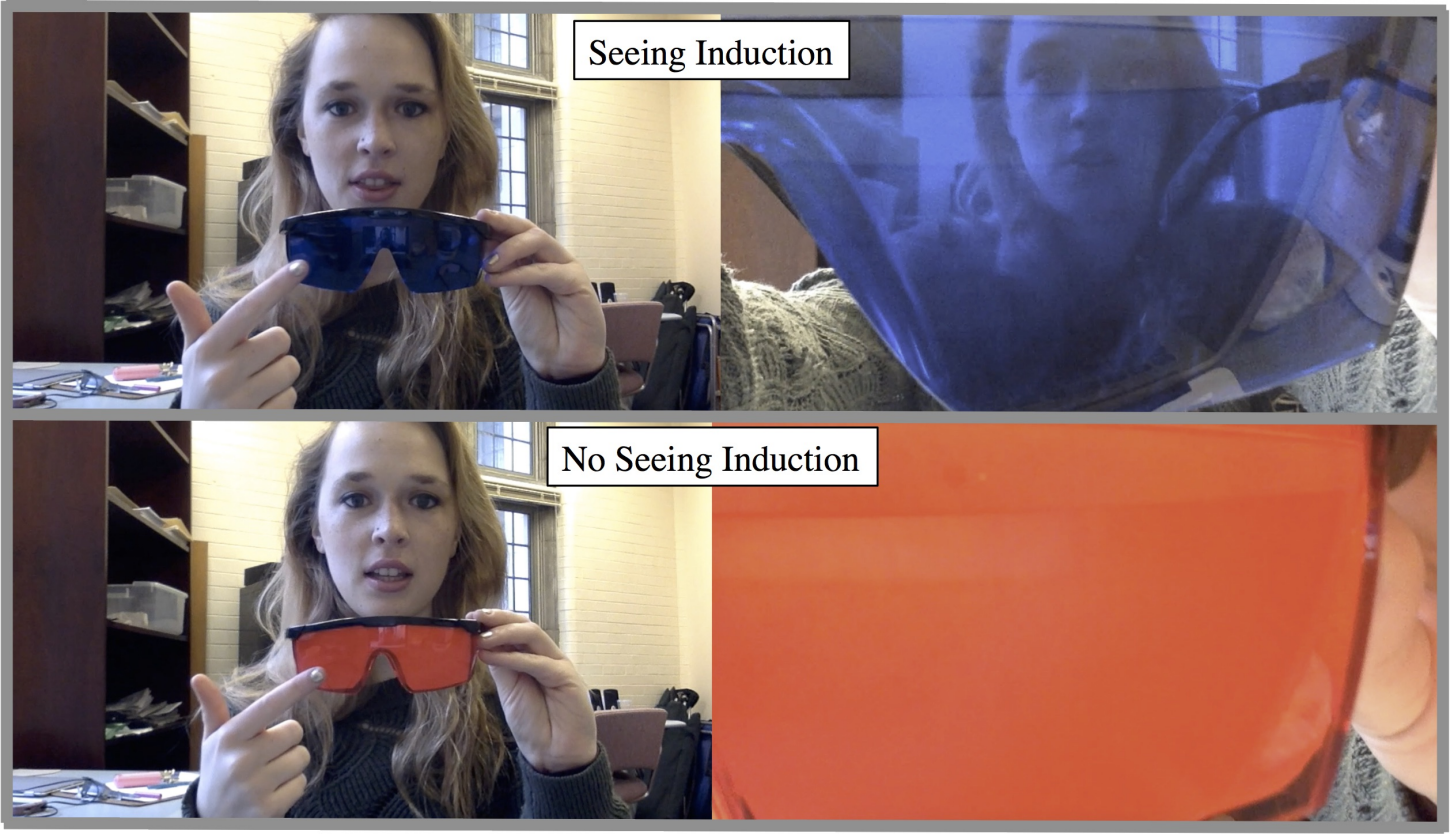
Depiction of belief induction event. The entire belief induction event was a video presented to participants online. The individual in this manuscript has given written informed consent (as outlined in PLoS consent form) to publish this image.

(2) Seeing induction. Participants next viewed a YouTube video (see sample video here: https://youtu.be/Yz80SYsDmgc; see Fig 3), which informed participants about which glasses—either red or blue—were transparent or opaque. Specifically, all participants saw a female experimenter reading the following script:

In this study, you will see a man standing in the middle of the room. In this room, there will be dots on the wall. Some of the time, the man will see less dots than you. And, other times you will see the same amount of dots as the man. It’s also important to note that sometimes the man will not be wearing any glasses at all. Other times, the man will be wearing red or blue glasses. Here’s the catch—the red and blue glasses are different. Here are the [blue/red] glasses. You can see—they are [blue/red]. When you wear these glasses, you cannot see through these glasses. Here are the [red/blue] glasses. You can see—they are [red/blue]. When you wear these glasses, you can see through these glasses. To repeat, here are the [blue/red] glasses. When you look through these glasses, you cannot see through them. Here are the [red/blue] glasses. When you look through these glasses, you can see through them.

Which glasses color were translucent and which were opaque were counterbalanced across participants. The experiment always held the glasses up to the screen to give the participant the experience of seeing through the glasses for both the translucent and opaque glasses.

After watching the belief induction video, participants had to respond to two comprehension questions. Specifically, they were shown a picture of an avatar in blue or red glasses (which color was depicted depended on which glasses were translucent). Here, the avatar saw 1 dot, whereas the participant saw 2. We asked, “If you were prompted with HIM and 1, what would you click: A or L? (Answer: A)” The second question depicted an avatar with blue or red glasses (which color was depicted dependent on which glasses were opaque). The avatar saw 1 dot, whereas the participant saw 3. Participants were asked, “If you were prompted with HIM and 1, what would you click: A or L (Answer: L). Participants had to respond correctly to these two questions to be included in final analyses. Participants were then directed to an external link to complete the experimental task.

(3) Practice trials. As in Study 1, participants first participated in 10 practice trials to adjust to the nature of the task and were told upon completing those practice trials that they were going to begin the experiment.

(4) Dot perspective-taking task. The dot perspective-taking task was based off of Furlanetto et al. [23]. Each trial consisted of a person standing in the middle of the room oriented either to the left or right. Across the trials, we manipulated whether the participant saw the same number of dots as the avatar (*consistency*: consistent, inconsistent), whether the participant was instructed to attend to their participant or the avatar (*perspective*: YOU, HIM), and whether the avatar could see (*seeing*: no goggles, translucent, opaque).

After manipulating consistency, perspective, and seeing in addition to randomizing which side of the screen the avatar looked, our sample included 288 trials. The participant and avatar saw the same number of dots on half of these trials t (i.e., consistent trials), whereas the participant saw more dots than the avatar on the other half of trials (i.e., inconsistent trials). On a third of the trials, the avatar wore no goggles. On another third, the avatar wore translucent goggles. On the last third, the avatar wore opaque goggles. These trials were then divided into six blocks each, and we also included two filler trials where no dots were displayed on the screen. We ensured that half of the test trials per block elicited “yes” responses, whereas the remaining trials elicited “no” responses. Translucent and opaque goggle trials were not intermixed within blocks as to avoid participant confusion. To ensure that each block comprised an equal number of trials, each block included 66% goggle trials and 33% no goggle trials. Block order and order within blocks were randomized for each participant.

**Fig 4:**
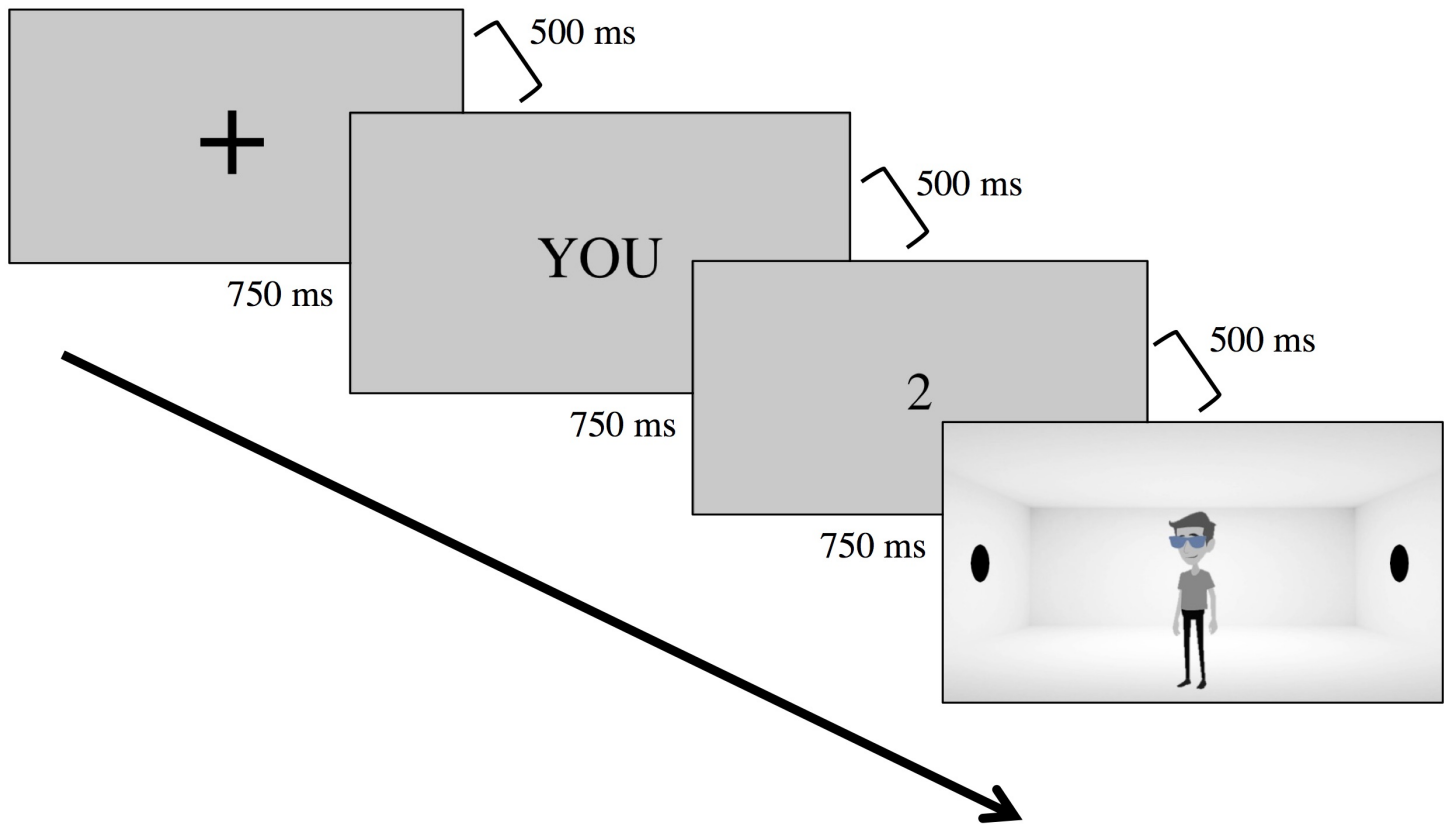
Depiction of Study 2’s procedure. Participants first saw a fixation cross followed by a perspective indication for either self (“YOU”) or other (“HIM”) trials. Participants then saw a number indication followed by an image of the avatar. At this point, participants then had 2000 ms to respond “yes” or “no” by clicking buttons on a computer.

The stimuli were programmed by and hosted on PsychPopUp (www.psychpopup.com). Participants first viewed a fixation cross for 750 ms and then viewed the perspective indication (YOU, HIM) for 750 ms (See Fig 4). There was a 500 ms pause in between the fixation cross and perspective indication. 500 ms after the perspective indication participants saw a number ranging from 0 to 3 proceeded by another 500 ms pause and then an image. The image remained on the screen for 2000 ms. Participants could respond yes or no during this 2000 ms interval. If participants did not respond during that period, the study then proceeded and the participant’s response was not included in analyses (1.31% of trials). Note that the dependent measure was not the sandbox measure utilized in Study 1, as we aimed to only replicate Furlanetto et al. [23] here.

### Results

#### Data preparation

We followed the same analytical strategy as Furlanetto et al. [23]. Only trials in which the participant answered correctly were included in response time analyses. If a participant’s response time on any given trial was 2.5 times the standard deviations of the participant’s mean response time, then the trial was not included in analyses. We also only examined trials for which participants responded “yes” because the “no” trials were disproportionately difficult in the inconsistent cases compared to the consistent cases. For all pairwise comparisons, we adopted a Bonferroni correction.

#### Response times

We conducted a 2 (Perspective: You, Him) x 2 (Consistency: Consistent, Inconsistent) x 3 (Seeing: No Goggles, Translucent Goggles, Opaque Goggles) repeated measures ANOVA. We found a significant main effect of Consistency, *F*(1, 33) = 24.10, *p* < .001, η_p_^2^ = .422. Participants took less time to respond in consistent trials, *M* = 861.40, *SD* = 211.82, compared to inconsistent trials, *M* = 893.58, *SD* = 208.71. We also found a significant main effect of Perspective, *F*(1, 33) = 18.67, *p* < .001, η_p_^2^ = .361. People took longer to respond when attending to the avatar’s perspective, *M* = 906.18, *SD* = 220.42, compared to when attending to their own perspective, *M* = 848.80, *SD* = 200.12. We did not find a significant main effect of Seeing, *F*(2, 32) = 2.22, *p* = .125, η_p_^2^ = .122, meaning participants did not taking any longer to respond across the three types of goggle trials (i.e., no goggles, translucent goggles, or opaque goggles).

We also found a Perspective x Consistency interaction, *F*(1, 33) = 13.31, *p* = .001, η_p_^2^ = .287. Participants took significantly longer to respond to “other” trials when the perspectives were inconsistent, *M* = 937.08, *SD* = 223.88, compared to consistent, *M* = 875.28, *SD* = 216.95; *t*(33) = 5.29, *p* < .001. These response times suggest that participants experienced egocentric interference such that they take longer to verify the avatar’s perspective because it conflicted with their own. Participants did not take any longer to respond to “self” trials when the perspectives were inconsistent, *M* = 847.53, *SD* = 206.70, compared to consistent ones, *M* = 850.08, *SD* = 193.54; *t*(33) = .25, *p* = .803. These response times suggest that participants did not experience altercentric interference.

We did not observe an interaction between Perspective and Seeing, *F*(2, 32) = 1.44, *p* = .25, η_p_^2^ = .082: participants did not take any longer to verify the “self” or “other” perspective indication across conditions. We did observe a marginally significant interaction between Consistency and Seeing, *F*(2, 32) = 3.30, *p* = .05, η_p_^2^ = .171. Participants took significantly longer to respond to the inconsistent trials in the no goggles condition, *M* = 906.39, *SD* = 211.01, and the translucent goggles condition, *M* = 915.35, *SD* = 233.20; *t*(33) = 5.10, *p* < .001, *t*(33) = 2.78, *p* = .009, respectively, compared to the consistent trials, *M* = 850.41, *SD* = 205.99; *M* = 880.11, *SD* = 225.91, respectively. There was no difference between consistent and inconsistent trials for the opaque goggles condition.

Critically, these two-way interactions were qualified by a marginally significant three-way interaction between Perspective, Consistency, and Seeing, *F*(2, 32) = 2.82, *p* = .075, η_p_^2^ = .150. Within the no goggles and translucent goggles condition, the Perspective x Consistency interaction was significant, *F*(1, 33) = 22.54, *p* = < .001, η_p_^2^ = .41; *F*(1, 33) = 6.67, *p* = .014, η^2^ = .17, respectively. The Perspective x Consistency interaction was not significant in the opaque goggles condition, however, *F*(1, 33) = .77, *p* = .386, η_p_^2^ = .02. As in Study 1, we examined the effect of gender. We did so because we realized that Furlanetto et al. [23] matched the gender of the avatar to the gender of the participant in case either factor would relate to implicit mentalizing. Contrary to this reasoning, we did not find an interaction effect of gender, *p* = .188.

**Fig 5:**
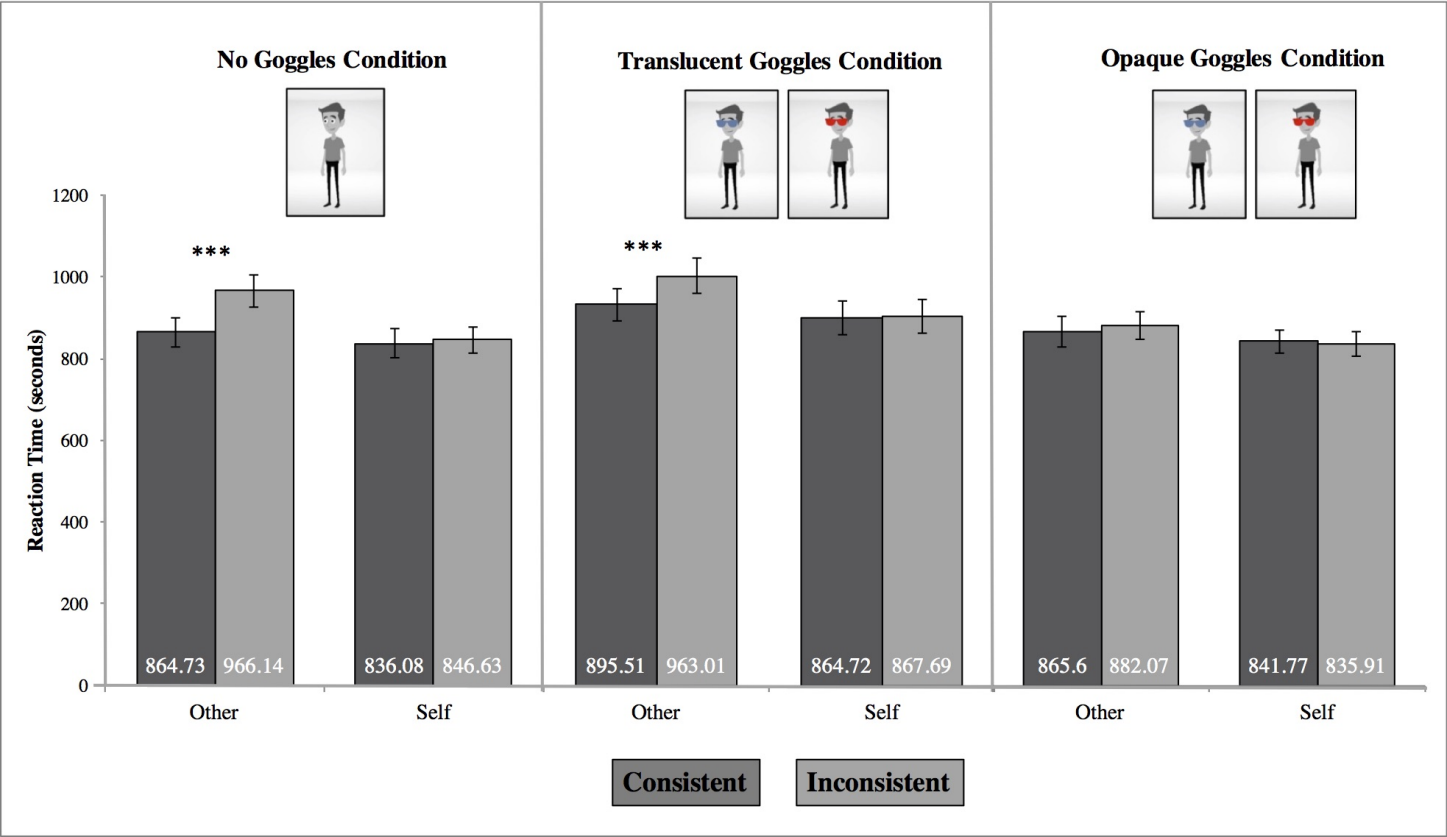
Depiction of Perspective x Consistency x Seeing interaction for response time measure. Error bars represent ±1 SEM; whether the translucent and opaque goggles were blue or red were counter-balanced across participants; *** *p* < .001.

We then examined pairwise comparisons within each seeing condition (See Table 1 for descriptive statistics). Doing so revealed that participants did not experience *altercentric* interference in any of the conditions: that is, participants took equally long to verify their own perspective in consistent and inconsistent trials for all of the seeing manipulations (all *p*s > .438; see Fig 5).

**Table 1:** Participants’ response times and errors in “self” and “other” trials.

	Response Times		Errors	
	Consistent Trials	Inconsistent Trials	Consistent Trials	Inconsistent Trials
**“Self” Trials** *[Altercentric Interference]*				
No goggles	836.08 (208.19)	846.63 (186.87)	.57 (.74)	1.23 (1.50)
Translucent goggles	864.72 (224.34)	867.69 (227.09)	.80 (1.08)	1.83 (1.63)
Opaque goggles	841.78 (187.59)	835.91 (166.66)	1.26 (.98)	1.37 (1.62)
**“Other” Trials** *[Egocentric Interference]*				
No goggles	864.73 (203.80)	966.14 (235.15)	.66 (.84)	1.94 (1.81)
Translucent goggles	895.51 (227.48)	963.01 (239.31)	1.26 (1.22)	2.00 (1.63)
Opaque goggles	865.60 (219.57)	882.07 (197.19)	2.14 (2.80)	1.66 (1.91)

Numbers not in parentheses represent mean values; numbers in parentheses represent standard deviations.

Participants did, however, exhibit *egocentric* interference, but only in the seeing and translucent goggles conditions. Specifically, participants took significantly longer to respond in the inconsistent trials compared to the consistent ones when asked about the avatar’s perspective in the no goggles and translucent goggles conditions, *t*(33) = 6.50, *p* < .001; *t*(33) = 4.15, *p* < .001, respectively (See Table 1). In the opaque goggles condition, however, participants did not exhibit egocentric interference, as there was no difference in people’s responses, *t*(33) = .69, *p* = .493.

### Errors

We conducted the same 2 (Perspective: You, Him) x 2 (Consistency: Consistent, Inconsistent) x 3 (Seeing: No Goggles, Translucent Goggles, Opaque Goggles) repeated measures ANOVA. We found a significant main effect of Consistency, *F*(1, 34) = 13.65, *p* = .001, η_p_^2^ = .29. Individuals made greater errors in the inconsistent condition, *M* = 1.67, *SD* = 1.68, compared to the consistent ones, *M* = 1.11, *SD* = 1.27. We also found a significant main effect of Perspective, *F*(1, 34) = 9.14, *p* = .005, η_p_^2^ = .21, whereby participants made greater egocentric errors on “other” trials, *M* = 1.61, *SD* = 1.70, than on “self” trials (i.e., altercentric errors), *M* = 1.18, *SD* = 1.26. There was also a significant main effect of Seeing, *F*(2, 33) = 16.60, *p* < .001, η_p_^2^ = .50. Participants made more errors in the opaque goggles condition, *M* = 1.61, *SD* = 1.83, and the translucent goggles condition, *M* = 1.47, *SD* = 1.39, compared to the no goggles condition, *M* = 1.10, *SD* = 1.29, *t*(34) = 3.60, *p* = .003; *t*(34) = 3.86, *p* = .001. The errors between the opaque and translucent goggles condition were not significantly different from one another (*p* = 1.00).

We also found a significant interaction between Consistency and Seeing conditions, *F*(2, 33) = 7.08, *p* = .003, η_p_^2^ = .30. Individuals made greater errors in the inconsistent trials, *M* = 1.91, *SD* = 1.63, compared to the consistent trials, *M* = 1.03, *SD* = 1.35, in the translucent condition, *t*(34) = 5.12, *p* < .001. Participants also made more errors in the inconsistent trials, *M* = 1.59, *SD* = 1.65, compared to consistent ones in the no goggles trials, *M* = .61, *SD* = .78; *t*(34) = 4.33, *p* < .001. However, participants did not make greater errors in the inconsistent trials, *M* = 1.51, *SD* = 1.77, compared to the consistent ones, *M* = 1.70, *SD* = 1.89, in the opaque condition. No other two-way interactions were significant.

**Fig 6:**
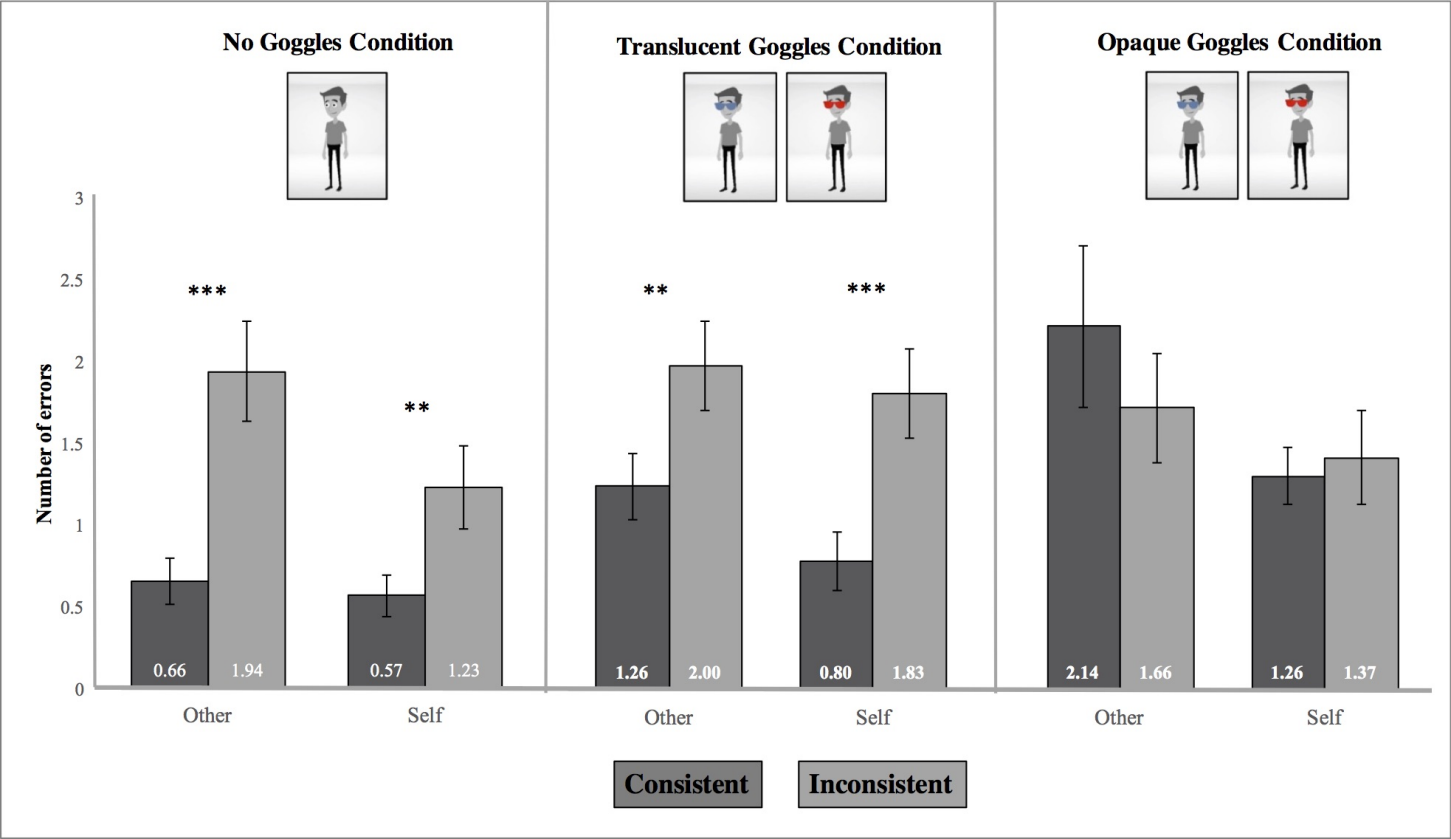
Depiction of Perspective x Consistency x Seeing interaction for error measure. Error bars represent ±1 SEM; whether the translucent and opaque goggles were blue or red were counter-balanced across participants; ** *p* < .01, *** *p* < .001.

We also found a significant Perspective x Consistency x Seeing interaction, *F*(2, 33) = 3.75, *p* = .034, η_p_^2^ = .19. We then examined the Perspective x Consistency interaction in the no goggles, translucent, and opaque conditions; none of them were significant, *F*(1, 34) = 2.83, *p* = .102, η_p_^2^ = .08; *F*(1, 34) = .51, *p* = .478, η_p_^2^ = .02; *F*(1, 34) = 1.12, *p* = .297, η_p_^2^ = .032, respectively (See Fig 6).

We then examined pairwise comparisons within each condition (See Table 1 for descriptive statistics). Doing so revealed that participants experienced *altercentric* interference in both the no goggles and translucent conditions, *t*(33) = 2.39, *p* = .022; *t*(33) = 3.63, *p* = .001, respectively; that is, participants made more errors in the inconsistent trials than the consistent ones in the “self” trials. We did not find a significant difference, however, in the opaque condition between the inconsistent and consistent trials in the “self” trials, *t*(33) = .39, *p* = .695. In other words, participants did not experience altercentric interference in this condition where the avatar was functionally blind.

Participants also exhibited *egocentric* interference in the no goggles and translucent goggles conditions. Specifically, participants made more errors in the inconsistent trials compared to the consistent ones in the “other” trials in the no goggles and translucent goggles conditions, *t*(33) = 4.18, *p* < .001; *t*(33) = 3.05, *p* = .004, respectively (See Table 1). In the opaque goggles condition, however, participants did not exhibit egocentric interference; that is, they did not make more errors in the inconsistent trials compared to the consistent ones.

### Discussion

Although we observed no evidence of altercentric interference in reaction time measures contrary to what has been found previously [23], we did find evidence of *altercentric* interference in participant error rates across perspective conditions. Specifically, participants made more errors when indicating their own perspective in inconsistent trials compared to consistent ones only in the trials where the avatar could see (i.e., when the avatar was not wearing goggles or wearing translucent ones). In the trials where the avatar could not see (i.e., the opaque goggle condition), no such effect was found. These findings provide support for the implicit mentalizing hypothesis and replicate the pattern that Furlanetto et al. [23] observed in response time findings at least when measured using error rates instead.

Participants’ response times, however, were not consistent with the possibility that participants experience altercentric interference. Participants did not take any longer to verify their own perspective between consistent and inconsistent trials in any of the conditions. These findings match the results of another replication attempt. Conway et al. [24] did not find the goggles manipulation to affect the degree of participants’ response times on “self” trials. In other words, Conway et al. [24] failed to find evidence of specific altercentric interference with respect to response times and accuracy measures.

With respect to *egocentric* interference, our findings mirror those of Furlanetto et al. [23] in both the error measure and also the response time measure. We document egocentric interference for both the no goggles and translucent goggles conditions, but not for the opaque goggle condition for both accuracy and response time.

## General discussion

The goal of the present studies was to explore whether adults rapidly represent the contents of other people’s mind. Specifically, we aimed to assess whether altercentric errors are socially specific [17, 18, 23] or domain-general [11, 12]. We did so by adopting a novel dependent measure with adults which has been shown to capture fine-grained representational accuracy differences in young children (Study 1), and by conducting a conceptual replication of a finding that we perceive as the best evidence to date that people do indeed implicitly model other’s perception, Furlanetto et al. [23] (Study 2).

In Study 1, we adopted a novel dependent measure, a “sandbox” measure of accuracy. We figured that this new measure would capture more fine-grained differences in the accuracy of participants’ representation of avatars compared to non-social controls. In doing so, however, we did not find support for the implicit mentalizing idea that adults rapidly represent the perspectives of human avatars differently than non-social arrows: participants exhibited altercentric interference on our accuracy measure for *both* arrows and avatars. In line with these findings, many other studies have found altercentric interference when the avatar was replaced with a less social stimulus [11–12].

These findings though can be explained in two ways. First, we may be seeing altercentric interference in both the avatar and the arrow condition because of strictly attentional cueing. Specifically, both the avatar and the arrow direct attention in a certain direction and that cueing could interfere with the participant’s judgments. Alternatively, it is also possible that the purportedly observed altercentric effects in the arrow condition are due to attentional cueing while the same effects in the avatar condition are due to implicit mentalizing. That is, although we see similar effects in both conditions, it is possible that the causes of these effects are different across the two conditions.

In light of this concern, we chose to conceptually replicate a study done by Furlanetto and colleagues [23]. This study was crafted in such a way that controlled for attentional cues across all conditions, yet was able to manipulate the avatar’s mental states by essentially “blinding” one of the avatars. In other words, the avatar was always facing a certain direction, but was functionally “blinded” by wearing opaque goggles in some trials. This method allowed us to discern whether altercentric errors are a function of simply attentional cueing or implicit mentalizing. The former account would predict that participants exhibit altercentric interference in all conditions, regardless of whether the avatar is blind or not. The latter account predicts that participants will show altercentric interference only in the conditions where the avatar can see.

Study 2 measured both participants’ response times and errors. We found disparate findings between the two measures. For response times, participants did not exhibit any altercentric interference in their response times—one critical effect on which the implicit mentalizing account depends. Furthermore, participants only exhibited egocentric interference for the avatar that could see, but not for the blinded avatar. Participants’ errors, however, were in line with the implicit mentalizing account insofar as they exhibited egocentric and altercentric errors only for the avatars who could see.

Collectively, the findings of Study 1 and 2 tentatively support an *intermediate* position regarding the nature of our capacities to implicitly and rapidly represent others’ perspectives. Researchers arguing for this intermediate position have noted that domain-general processes, such as attentional cueing, may play some role in domain-specific theory of mind processes [30]. Furthermore, this position also argues that merely imputing someone’s perspective may involve a domain-general directional cue, and thus similar effects in non-social and social cases do not necessarily mean that participants fail to represent the contents of individuals’ minds entirely.

Our findings may shed light on the specific relationship between perspective-taking and attention cuing mechanisms in implicit mentalizing tasks. We found that participants purportedly expressed altercentric interference for an arrow but not for a blind avatar. These findings suggest that, in the case of the blind avatar, attentional cues are not activated or that perspective-taking mechanisms subsume any attentional cueing effects. Indeed, Michael and colleagues [31] recently found that perspective-taking processes are recruited *earlier* than attention cueing ones. Because of this temporal difference, it is possible that participants rapidly ignored the perspective of the blind avatar and thus attentional cueing mechanisms were not subsequently activated. Or if they were, they were then ignored. For the arrow, however, perspective-taking processes were never recruited because the arrow is not human, and thus attentional cues gave rise to effects that look like altercentric interference effects but are instead not a function of rapid mentalizing. Ultimately though, future research is needed to assess precisely why it is that different altercentric effects emerge on different tasks and measures in addition to in various contexts.

### Limitations and lingering questions

Beyond this intermediate account though, there are also methodological reasons why we may have not documented heightened altercentric interference in the avatar condition compared to the arrow condition in Study 1. For example, it is possible that we did not find varying altercentric interference effects because we mixed social and non-social trials in the experiment. Although previous work has documented socially specific altercentric interference effects in mixed designs [20, 23], it is certainly plausible that interspersing trials may make it more challenging to detect differences between social and non-social trials. Whether such a change would elicit different results in Study 1 is a potential next step to examine the socially specified nature of theory of mind effects.

Second, it is also worth noting that Study 1 deviated from the typical dot perspective-taking tasks beyond the implementation of a new, continuous accuracy measure. Specifically, the avatar saw more than 3 dots in our paradigm; in other work, the number of dots was limited to 3 to ensure the number of dots were within the subitizing range. Interestingly, we did not find any differences across the different number trials. One explanation for this may be that the task was recruiting non-automatic cognitive resources. We find this explanation unlikely, however, considering participants made systematic errors in the task in line with previous findings suggesting participants were not relying exclusively on explicit processing throughout the task, but future research could better examine this possibility.

Third, another methodological consideration lies with the “sandbox” accuracy measure. Although we adopted this measure as a means of capturing representational variance in a more fine-grained way, it is plausible that the measure was not sensitive enough to capture small differences between social and non-social trials. Perhaps this is because participants in the task were not making errors of an egregious magnitude (errors ranged from -1.07 to .24), even though the number line lacked discrete number labels. Participants’ accuracy suggests that socially specified altercentric errors may be difficult to detect in accuracy measures because the task is fairly easy for participants in general.

Finally, it is also possible that the measure did not recruit implicit processing because the task required more than simply responding yes or no and instead required participants to move a mouse to click on a number line. It seems unlikely though that the task used in Study 1 failed to recruit implicit processing when considering evidence suggesting that tasks that require quick mouse movements under time pressure capture implicit processing [32]. Still, future research could consider adopting different measures of accuracy, including eye tracking or mouse tracking methodologies [33], when assessing the social nature of implicit theory of mind effects.

With respect to Study 2, we documented socially specific altercentric interference with error measures but not with response times. Furlanetto and colleagues [23] documented these effects with response times but not with errors (although participants’ errors resembled their response times). Why is this the case? The major difference between our study and Furlanetto and colleagues’ [23] study was that ours was conducted online, whereas their study was conducted in the lab. It is possible that response time measures are higher (i.e., slower) in online experiments on Amazon Mechanical Turk, as the experimental context is much less controlled and participants may be susceptible to a variety of factors that are not present in the lab. Indeed, the response times we document in the current paper are longer than those documented in Furlanetto et al’s [23] work.

Notably, longer response times may suggest more deliberative rather than implicit responding. This is unlikely to be the case, however, because we did not find any evidence of a speed-accuracy trade off; none of the correlations between speed and accuracy were significant. Furthermore, if participants were responding deliberately, they should not have exhibited differentiating error rates depending on condition. Finally, with respect to differences in error findings between our data and those of Furlanetto and colleagues [23]: We tested additional participants compared to this original study and therefore had larger power to detect altercentric errors.

Aside from being conducted online, Study 2’s method did slightly vary from that of Furlanetto et al. [23]’s study. The avatar used in our images in the present studies was not directly facing the side walls as in the original study. Instead, the avatar’s body was oriented slightly more toward the front while his head shifted to either the left or right. It is plausible that participants may have had more difficulty computing the avatar’s line-of-sight, which then shifted participants to rely on more explicit processing to perform the task. Prior to performing the main task, however, participants did have to pass a set of comprehension checks that relied on participants assuming that the avatar could see all the dots or squares on the wall.

### Conclusion

Taken together with other conflicting findings, the present results demonstrate the importance of replications within the domain of implicit mentalizing effects (see also [10]). This need is especially pertinent considering that assessing whether individuals possess a domain-specific implicit system is critical to characterizing the nature of humans’ theory of mind capacities [4]. In two studies, we provide evidence in favor of the account that attentional cues and theory of mind processes both matter for representing the contents of other people’s minds. That is, altercentric interference effects tested using accuracy measures emerge for both arrows and avatars (Study 1), but not for blinded avatars (Study 2). Overall, the current findings contribute to a greater conversation regarding whether individuals implicitly model other people’s perception. Future research should focus on exactly how attentional cueing and theory of mind processes interact and complement one another.

## Supporting information

S1 File Study 1 reaction time data. (DOCX)

## References

[1] Onishi KH, Baillargeon R. Do 15-month-old infants understand false beliefs?. Science. 2005;308:255–258. doi: 10.1126/science.1107621 15821091 10.1126/science.1107621 PMC3357322

[2] Sodian B, Thoermer C, Metz U. Now I see it but you don't: 14-month-olds can represent another person's visual perspective. Developmental Science. 2007;10:199–204. doi: 10.1111/j.1467-7687.2007.00580.x 17286844 10.1111/j.1467-7687.2007.00580.x

[3] Apperly IA, Butterfill SA. Do humans have two systems to track beliefs and belief-like states?. Psychological Review. 2009;116:953–970. doi: 10.1037/a0016923 19839692 10.1037/a0016923

[4] Butterfill SA, Apperly IA. How to construct a minimal theory of mind. Mind & Language. 2013;28:606–637.

[5] Kovács ÁM, Téglás E, Endress AD. The social sense: Susceptibility to others’ beliefs in human infants and adults. Science. 2010;330:1830–1834. doi: 10.1126/science.1190792 21205671 10.1126/science.1190792

[6] Wellman HM, Cross D, Watson J. Meta-analysis of theory-of-mind development: the truth about false belief. Child Development. 2001;72:655–684. 11405571 10.1111/1467-8624.00304

[7] Schneider D, Slaughter VP, Bayliss AP, Dux PE. A temporally sustained implicit theory of mind deficit in autism spectrum disorders. Cognition. 2013;129:410–417. doi: 10.1016/j.cognition.2013.08.004 23994318 10.1016/j.cognition.2013.08.004

[8] Senju A, Southgate V, White S, Frith U. Mindblind eyes: an absence of spontaneous theory of mind in Asperger syndrome. Science. 2009;325:883–885. doi: 10.1126/science.1176170 19608858 10.1126/science.1176170

[9] Schneider D, Slaughter VP, Dux PE. Current evidence for automatic Theory of Mind processing in adults. Cognition. 2017;162:27–31. doi: 10.1016/j.cognition.2017.01.018 28189035 10.1016/j.cognition.2017.01.018

[10] Phillips J, Ong DC, Surtees AD, Xin Y, Williams S, Saxe R, Frank MC. A second look at automatic theory of mind: Reconsidering Kovács, Téglás, and Endress (2010). Psychological Science. 2015;26:1353–1367. doi: 10.1177/0956797614558717 26253550 10.1177/0956797614558717

[11] Santiesteban I, Catmur C, Hopkins SC, Bird G, Heyes C. Avatars and arrows: Implicit mentalizing or domain-general processing?. Journal of Experimental Psychology: Human Perception and Performance. 2014;40:929–937. doi: 10.1037/a0035175 24377486 10.1037/a0035175

[12] Heyes C. Submentalizing: I am not really reading your mind. Perspectives on Psychological Science. 2014;9:131–143. doi: 10.1177/1745691613518076 26173251 10.1177/1745691613518076

[13] Lin S, Keysar B, Epley N. Reflexively mindblind: Using theory of mind to interpret behavior requires effortful attention. Journal of Experimental Social Psychology. 2010;46:551–556.

[14] Samson D, Apperly IA, Braithwaite JJ, Andrews BJ, Bodley Scott SE. Seeing it their way: Evidence for rapid and involuntary computation of what other people see. Journal of Experimental Psychology: Human Perception and Performance. 2010;36:1255–1266. doi: 10.1037/a0018729 20731512 10.1037/a0018729

[15] Qureshi AW, Apperly IA, Samson D. Executive function is necessary for perspective selection, not Level-1 visual perspective calculation: Evidence from a dual-task study of adults. Cognition. 2010;117:230–236. doi: 10.1016/j.cognition.2010.08.003 20817158 10.1016/j.cognition.2010.08.003

[16] Birch SA, Bloom P. Understanding children's and adults' limitations in mental state reasoning. Trends in Cognitive Sciences. 2004;8:255–260. doi: 10.1016/j.tics.2004.04.011 15165550 10.1016/j.tics.2004.04.011

[17] Nielsen MK, Slade L, Levy JP, Holmes A. Inclined to see it your way: Do altercentric intrusion effects in visual perspective taking reflect an intrinsically social process?. The Quarterly Journal of Experimental Psychology. 2015;68:1931–1951. doi: 10.1080/17470218.2015.1023206 25849956 10.1080/17470218.2015.1023206

[18] Drayton LA, Boothby EJ, Santos LR, & Dunham Y. Race affects visual perspective taking. In prep.

[19] Cole GG, Atkinson M, Le AT, Smith DT. Do humans spontaneously take the perspective of others?. Acta Psychologica. 2016;164:165–168. doi: 10.1016/j.actpsy.2016.01.007 26826864 10.1016/j.actpsy.2016.01.007

[20] Baker LJ, Levin DT, Saylor MM. The extent of default visual perspective taking in complex layouts. Journal of Experimental Psychology: Human Perception and Performance. 2016;42:508–516. doi: 10.1037/xhp0000164 26551519 10.1037/xhp0000164

[21] Santiesteban I, Shah P, White S, Bird G, Heyes C. Mentalizing or submentalizing in a communication task? Evidence from autism and a camera control. Psychonomic Bulletin & Review. 2015;22:844–849. 25149442 10.3758/s13423-014-0716-0

[22] Wilson CJ, Soranzo A, Bertamini M. Attentional interference is modulated by salience not sentience. Acta Psychologica. 2017;178:56–65. doi: 10.1016/j.actpsy.2017.05.010 28578296 10.1016/j.actpsy.2017.05.010

[23] Furlanetto T, Becchio C, Samson D, Apperly I. Altercentric interference in level 1 visual perspective taking reflects the ascription of mental states, not submentalizing. Journal of Experimental Psychology: Human Perception and Performance. 2016;42:158–163. doi: 10.1037/xhp0000138 26389611 10.1037/xhp0000138

[24] Conway JR, Lee D, Ojaghi M, Catmur C, Bird G. Submentalizing or mentalizing in a Level 1 perspective-taking task: A cloak and goggles test. Journal of Experimental Psychology: Human Perception and Performance. 2017; 43:454–465. doi: 10.1037/xhp0000319 27893269 10.1037/xhp0000319 PMC5327864

[25] Bernstein DM, Thornton WL, Sommerville JA. Theory of mind through the ages: Older and middle-aged adults exhibit more errors than do younger adults on a continuous false belief task. Experimental Aging Research. 2011;37:481–502. doi: 10.1080/0361073X.2011.619466 22091578 10.1080/0361073X.2011.619466

[26] Sommerville JA, Bernstein DM, Meltzoff AN. Measuring beliefs in centimeters: Private knowledge biases preschoolers' and adults' representation of others' beliefs. Child Development. 2013;84:1846–1854. doi: 10.1111/cdev.12110 23581849 10.1111/cdev.12110

[27] Wimmer H, Perner J. Beliefs about beliefs: Representation and constraining function of wrong beliefs in young children's understanding of deception. Cognition, 1983;13:103–128. 6681741 10.1016/0010-0277(83)90004-5

[28] Cantlon JF, Brannon EM. Shared system for ordering small and large numbers in monkeys and humans. Psychological Science. 2006;17:401–406. doi: 10.1111/j.1467-9280.2006.01719.x 16683927 10.1111/j.1467-9280.2006.01719.x

[29] Wagenmakers EJ. A practical solution to the pervasive problems of p values. Psychonomic Bulletin & Review. 2007;14: 779–804. 18087943 10.3758/bf03194105

[30] Michael J, D’Ausilio A. Domain-specific and domain-general processes in social perception–A complementary approach. Consciousness and Cognition. 2015; 36: 434–437. doi: 10.1016/j.concog.2014.12.009 25555326 10.1016/j.concog.2014.12.009

[31] Michael JM, Wolf T, Letesson C, Butterfill S, Skewes J, Hohwy J. Seeing it both ways: Using a double-cuing task to investigate the role of spatial cuing in level-1visual perspective-taking. Journal of Experimental Psychology: Human Perception and Performance. 2017. 10.1037/xhp0000486 29154620

[32] Freeman JB, Johnson KL. More than meets the eye: split-second social perception. Trends in Cognitive Sciences. 2016; 20;362–374. doi: 10.1016/j.tics.2016.03.003 27050834 10.1016/j.tics.2016.03.003 PMC5538856

[33] van der Wel RP, Sebanz N, Knoblich G. Do people automatically track others’ beliefs? Evidence from a continuous measure. Cognition. 2014;130: 128–133. doi: 10.1016/j.cognition.2013.10.004 24216021 10.1016/j.cognition.2013.10.004

