# Supplementary material for: Does altercentric interference rely on mentalizing?: Results from two level-1 perspective-taking tasks
Source: PLoS One. 2018 Mar 22;13(3):e0194101. doi: 10.1371/journal.pone.0194101 (PMC5864002; doi:10.1371/journal.pone.0194101)
Supplement: S1 File — (DOCX) [file pone.0194101.s001.docx]

**S1 File** – Study 1 Reaction Time Data

In addition to the accuracy measures we report in the main text, we also analyzed participants’ reaction times to see whether their responses would clarify any of the findings from the accuracy measure. We did so in an exploratory fashion, however, considering Study 1’s task was not designed to capture differences in individuals’ reaction times. Thus, we first conducted a 2 (Perspective: You, Him) x 2 (Consistency: Consistent, Inconsistent) x 2 (Sociality: Human, Arrow) x 2 (Ratio: Small, Large) repeated measures ANOVA. Here there was a significant Perspective x Consistency x Sociality x Ratio interaction, *F*(1, 48) = 5.04, *p* = .039, η_p_^2^ = .095. We examined the Perspective x Consistency x Sociality interaction for both small and large ratios. This interaction was not significant for small ratios, *p* = .139, and was marginally significant for the large ratios, *p* = .088. Looking at pairwise comparisons utilizing a Bonferroni correction revealed that participants in the large ratio condition expressed an altercentric and egocentric effect for only the arrow condition, *p* = .051 for “YOU” trials (i.e., altercentric interference) and *p* = .006 for “HIM” trials (i.e., egocentric interference). Participants did not express altercentric or egocentric interference on any of the other trials.

We then collapsed across ratios given that we were primarily interested in how the sociality manipulation affected reaction times. In this analysis, the Perspective x Consistency x Sociality interaction was not significant, *F*(1, 48) = .19, *p* = .668, η_p_^2^ = .004. Collapsing across sociality, we also did not find a significant Perspective x Consistency interaction, *F*(1, 48) = .1.55, *p* = .219, η_p_^2^ = .031. These findings suggest that reaction times in this task did not track egocentric and altercentric interference effects in the same way as reaction times generally do in the canonical dot perspective-taking task. Instead, what better revealed the implicit mentalizing effect was the accuracy measure for which we planned to be the primary dependent variable of interest.
